# *OsTIR1* and *OsAFB2* Downregulation via *OsmiR393* Overexpression Leads to More Tillers, Early Flowering and Less Tolerance to Salt and Drought in Rice

**DOI:** 10.1371/journal.pone.0030039

**Published:** 2012-01-10

**Authors:** Kuaifei Xia, Ren Wang, Xiaojin Ou, Zhongming Fang, Changen Tian, Jun Duan, Yaqin Wang, Mingyong Zhang

**Affiliations:** 1 Key Laboratory of South China Agricultural Plant Genetics and Breeding, South China Botanical Garden, Chinese Academy of Sciences, Guangzhou, China; 2 Key Laboratory of Plant Resources Conservation and Sustainable Utilization, South China Botanical Garden, Chinese Academy of Sciences, Guangzhou, China; 3 Graduate University of Chinese Academy of Sciences, Beijing, China; 4 School of Life Science, Guangzhou University, Guangzhou, China; 5 Guangdong Key Lab of Biotechnology for Plant Development, College of Life Science, South China Normal University, Guangzhou, China; East Carolina University, United States of America

## Abstract

The microRNA *miR393* has been shown to play a role in plant development and in the stress response by targeting mRNAs that code for the auxin receptors in *Arabidopsis*. In this study, we verified that two rice auxin receptor gene homologs (*OsTIR1* and *OsAFB2*) could be targeted by *OsmiR393* (*Os* for *Oryza sativa*). Two new phenotypes (increased tillers and early flowering) and two previously observed phenotypes (reduced tolerance to salt and drought and hyposensitivity to auxin) were observed in the *OsmiR393*-overexpressing rice plants. The *OsmiR393*-overexpressing rice demonstrated hyposensitivity to synthetic auxin-analog treatments. These data indicated that the phenotypes of *OsmiR393*-overexpressing rice may be caused through hyposensitivity to the auxin signal by reduced expression of two auxin receptor genes (*OsTIR1* and *OsAFB2*). The expression of an auxin transporter (*OsAUX1*) and a tillering inhibitor (*OsTB1*) were downregulated by overexpression of *OsmiR393*, which suggested that a gene chain from *OsmiR393* to rice tillering may be from *OsTIR1* and *OsAFB2* to *OsAUX1*, which affected the transportation of auxin, then to *OsTB1*, which finally controlled tillering. The positive phenotypes (increased tillers and early flowering) and negative phenotypes (reduced tolerance to salt and hyposensitivity to auxin) of *OsmiR393*-overexpressing rice present a dilemma for molecular breeding.

## Introduction

Plant architecture, fertility and flowering time are important in the genetic improvement of rice [1]. These traits are generally controlled by multiple genes and are influenced by the environment. Recently, plant microRNAs (miRNAs) have been shown to play important regulatory roles in plant development and response to environmental stresses by targeting mRNAs for mRNA cleavage, mRNA decay or translational repression [2], [3]; therefore, they may be useful for molecular breeding.

Many miRNAs regulate organ development from embryo to leaves in plants [2], [4]. In rice, *OsSPL14* controls plant architecture and affects grain yield, and it is regulated by *OsmiR156* [1]. Upregulation of *OsmiR172* induced a loss of spikelet determinacy and floral organ abnormalities in rice [5]. Many miRNAs also regulate phase changes in plants [2], [6]. *Arabidopsis miR172*, *miR159*, *miR156* and *miR171* regulate flowering time and floral patterning [2], [7], [8]. Many miRNAs are involved in the stress responses of plants against different environmental factors [3], [9], [10], [11] and also have important roles in plant signal transduction systems (such as *miR393* and *miR167*) [12], [13], [14] and the production of *Ta-siRNA*, an endogenous trans-acting siRNA [15].

Many microRNA gene families are evolutionarily conserved across all major plant lineages [16]. As one of the conserved miRNA families in plants, *miR393* genes have been found in different plant species including rice [4], [17], [18]. Although miRNA precursors (pre-miRNAs) of *miR393* vary with plant species, the length (21 bp) and the sequence of the mature miRNA are conserved among different species [4], [17]. The microRNA *miR393* has been shown function via the auxin pathway by posttranscriptional regulation of auxin receptors [4], [12], [17]. In *Arabidopsis*, *miR393* targets mRNAs that code for the auxin receptors (*TIR1*, *AFB2* and *AFB3*) [4], [12]. The microRNA *miR393* has been found to have a role in leaf development [4] and normal development [14] and in response to pathogen attack [12] and salt stress [17], [18], [19]. In rice, *OsmiR393* was recently found to play an important role in response to salt stress [17].

However, the targeting genes of *OsmiR393* have not been determined in rice. Apart from the response to salt, other effects of *OsmiR393* on rice growth and development are still unknown, as is whether it is a potential target for rice molecular breeding. We constructed *OsmiR393* overexpressing transgenic rice to answer these questions. Our results indicated that *OsmiR393* could target two rice auxin receptor genes (*OsTIR1* and *OsAFB2*), and two new functions were identified through *OsmiR393* overexpression. An increase in tillers and early flowering were caused by overexpression of *OsmiR393*, in addition to a decreased tolerance to salt and hyposensitive to auxin.

## Results

### 1. Seven rice genes were predicted to be putative target genes of *OsmiR393*

To identify the potential target genes of *OsmiR393*, the web-based prediction program miRU (Plant microRNA Potential Target Finder, http://bioinfo3.noble.org/miRNA/miRU.htm) was used, and seven candidate genes were obtained from the rice genome. Information regarding these seven genes is listed in Table 1. There were at least three mismatch nucleotides in the target sites of the seven mRNAs compared to the 21 nucleotides of *OsmiR393*. Of the seven candidate genes, the functions of five genes have been predicted; however, the functions of LOC_Os04g58734 and LOC_Os03g36080 have not been predicted. Two genes (LOC_Os04g32460 and LOC_Os05g05800) were highly homologous with the auxin receptors *OsAFB2* and *OsTIR1*, respectively; the similarity of *OsAFB2* to *Arabidopsis AFB2* and *OsTIR1* to *Arabidopsis TIR1* were 80% and 77%, respectively (Table 1). The other three genes (LOC_Os03g52320, LOC_Os10g39790 and LOC_Os05g41010) showed significant similarity to the GRF1-interacting factor, magnesium transporter and prolyl-4-hydroxylase, respectively.

**Table 1:** Putative target genes for *OsmiR393* in rice predicted based on the sequence complementarity.

Gene ID	Gene name	Description	Similarity (%)	Full-length cDNA	Target site alignment^a^	Actual target
	*OsmiR393*				3′CCUAGUUACGCUAGGGAAACCU5′	
LOC_Os04g32460	*OsAFB2*	Auxin signaling f-box 2	80.38 (At3g26810)	AK072338 (*OsAFB2-1*) AK100862 (*OsAFB2-2*)	5′G**AGA**CAAUGCGAUCCCUUUGGA3′	Yes
LOC_Os04g58734				AK060126 AK066367	**C**GAUCA**G**UGC**A**AUCCCUUUGGA	No
LOC_Os05g05800	*OsTIR1*	Protein transport inhibitor response (auxin receptor)	76.82 (At3g62980)		G**AGA**CAAUGCGAUCCCUUUGGA	Yes
LOC_Os03g36080					**CAG**UC**U**A**C**GCGAUCCCUUUGGA	No
LOC_Os03g52320	*OsGRF1*	GRF1-interacting factor 3	54.92 (At4g00850)	AK058575	**U**G**C**U**G**AAUGCGAUC**U**CUUUGG**G**	No
LOC_Os10g39790	*OsMGT6*	Magnesium transporter	68.81 (At3g58970)	AK069472	**C**GAUC**G**AUG**U**GAUCCCUUU**U**G**U**	No
LOC_Os05g41010		Prolyl-4-hydroxylase	81.61 At5g18900		**UU**AUCA**G**UGC**A**AU**UU**CUUUGGA	No

The putative target genes for *OsmiR393* were predicted using the web-based program (http://bioinfo3.noble.org/miRNA/miRU.htm).

a Mismatch nucleotides in target mRNAs with *OsmiR393* are underlined.

### 2. Overexpression of *OsmiR393* reduced the expression levels of two auxin receptor gene homologs

**Figure 1:**
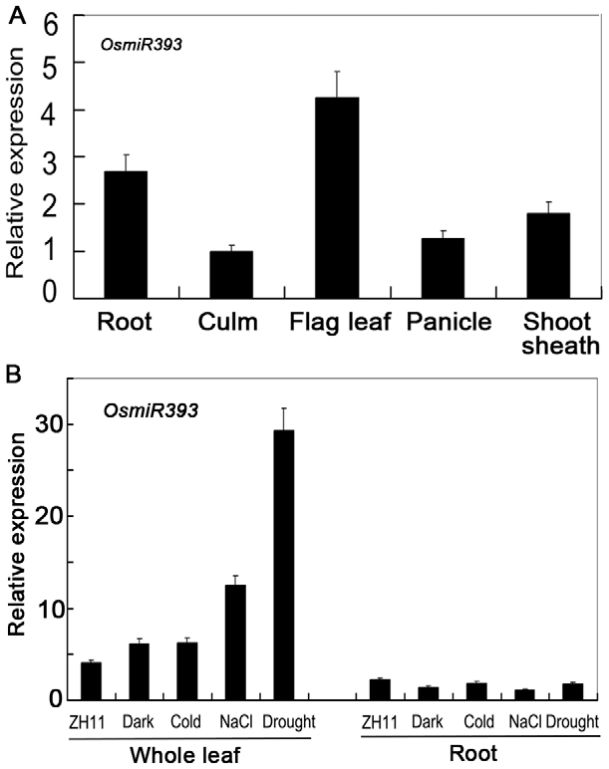
Relative expression levels of *OsmiR393* in different organs under normal growth conditions and under abiotic stresses. Expression levels of *OsmiR393* were normalized to the reference gene snRNA *U6* [48], whose expression level was defined as 1. **A.** RNA was extracted from the booting rice plants, which were grown in the natural field. **B.** Expression levels of *OsmiR393* in response to abiotic stress. For salt treatment, the seedlings were kept in a 250 mM NaCl solution for 4 h. For drought treatment, the seedlings were dried for 4 h between folds of tissue paper at 28±1°C. For cold treatment, the seedlings were kept at 4±1°C for 4 h. For dark treatment, seedlings were kept in the dark for 48 h.

**Figure 2:**
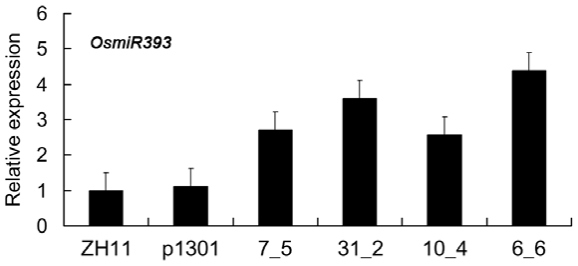
Verification of the 22-bp mature-*OsmiR393* overexpression in *OsmiR393*-overexpressing transgenic rice lines by real-time RT-PCR. RNA was extracted from the flag leaves of the second generation of the transgenic rice plants. The expression level of *OsmiR393* in wild-type rice ZH11 was set as one; p1301 is the transgenic ZH11 with the empty vector. The *OsmiR393*-overexpressing transgenic lines 7_5 and 31_2 are homozygous, and the lines 10_4 and 6_6 are heterozygous. These lines are the same as in Figures 3–10; snRNA *U6* [48] served as a reference gene for the detection of miRNAs.

To investigate whether *OsmiR393* could target the above predicted genes, we generated transgenic rice plants that overexpress *OsmiR393*. Expression of the DNA sequence containing the mature folded structure of *OsmiR393* was driven by the constitutive 35S promoter. This construct was introduced into the rice variety Zhonghua 11 through *Agrobacterium*-mediated transformation [20]. Sixty independent transgenic plant lines were confirmed to have successfully integrated *35S:OsmiR393* into the rice genome using PCR amplification with the pair of primers O27F and G4369; 28 lines exhibited overexpression of *OsmiR393*. When the plants were grown in a natural paddy, q-RT-PCR results showed that the expression levels of *OsmiR393* was the highest in the roots and flag leaves among the five organs tested at the booting stage (Figure 1A). In addition, the expression of *OsmiR393* was significantly upregulated by salt and drought in the leaves, but not in the roots (Figure 1B), when using at least a two-fold threshold for expression changes. Our experiments also verified that the expression of *OsmiR393* in four independent transgenic lines was stably upregulated in the flag leaves of the first (T_1_) (data not shown) and the second (T_2_; Figure 2) generation of transgenic plants. These indicated that the *OsmiR393* transgene is inherited persistently in rice; therefore, these four transgenic lines were used in subsequent experiments. The expression of *OsmiR393* in the four transgenic rice plants was upregulated at least by two fold compared to the two controls, which were the wild-type rice variety Zhonghua 11 (ZH11) and the transgenic rice plants with empty vector pCambia1301 (Figure 2).

**Figure 3:**
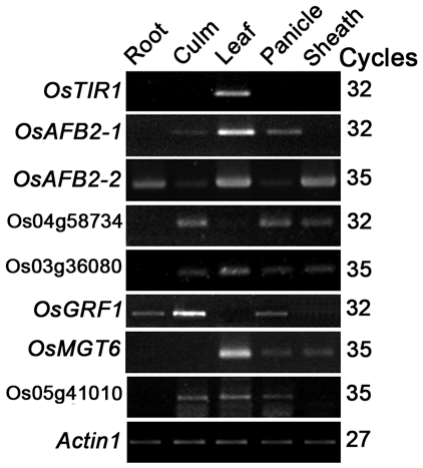
Organ-specific expression analysis for the predicted target genes of *OsmiR393* by semi-quantitative RT-PCR. Total RNA was isolated from booting rice plants grown in the natural field. Rice *Actin1* was used as an internal control. *OsAFB2-1* and *OsAFB2-2* are two alternatively spliced mRNAs of *OsAFB2*. The term “cycles” indicates the PCR cycle number used in RT-PCR reactions.

**Figure 4 pone-0030039-g004:**
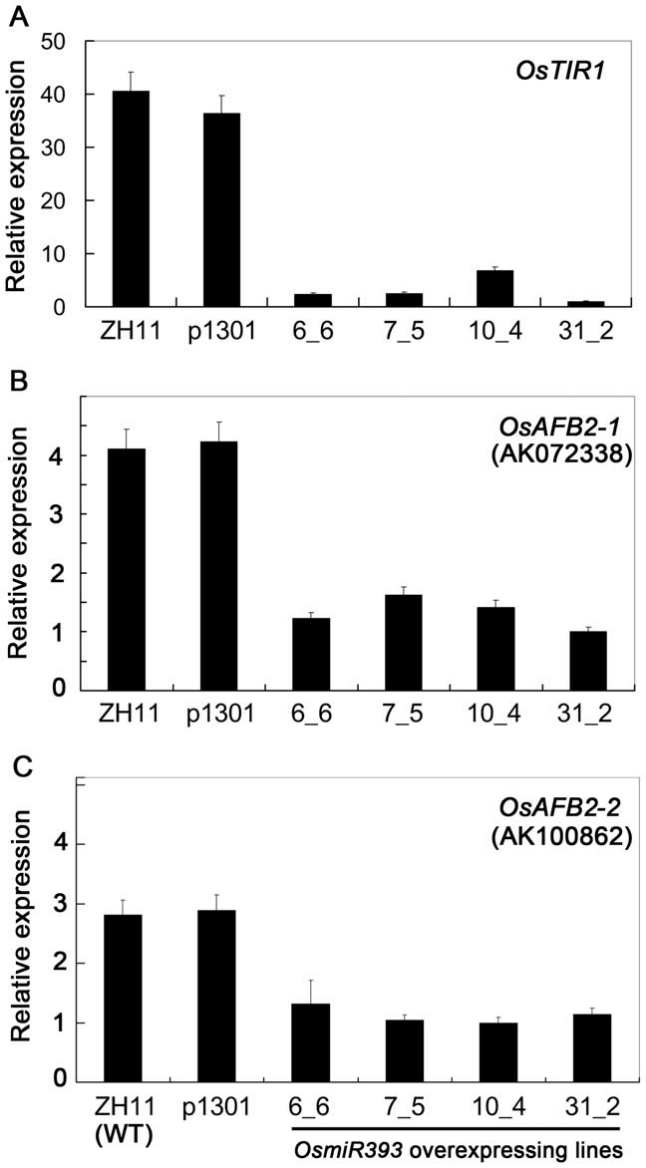
Expression identification of the predicted target mRNAs in *OsmiR393*-overexpressing transgenic lines. Transcript levels of the target mRNAs were measured by real-time RT-PCR when the rice plants were grown in the natural field. The plots represent the relative expression (fold) of each gene in the transgenic plants compared with the expression of *Actin1*. Mean values and standard errors were obtained from three independent experiments.

To confirm whether the transcripts of the seven putative target genes were downregulated in the transgenic plants overexpressing *OsmiR393*, real time-RT-PCR was performed to measure the transcript abundance of the seven putative target genes using the primer pairs listed in Table S1. We first investigated the organ expression of these candidates by semi-quantitative RT-PCR (Figure 3). Then, we used the organs that showed the highest expression for the corresponding candidate genes to monitor gene expression in the *OsmiR393*-overexpressing rice plants. Among the seven candidate genes, only two genes (*OsAFB2*: LOC_Os04g32460 and *OsTIR1*: LOC_Os05g05800) were repressed in the homozygous and heterozygous lines of the T_1_ and T_2_ *OsmiR393*-overexpressing rice plants (Figure 4). To detect actual cleavage of the candidate genes in the *OsmiR393*-overexpressing lines, 5′RACE was used to monitor the cut fragments of the mRNAs of *OsAFB2* and *OsTIR1*; 5′RACE detected the expected cleavage fragments of *OsAFB2* and *OsTIR1* mRNAs in the *OsmiR393* over-expressing rice plants (Figure S1). These data suggested that *OsmiR393* could target the *OsAFB2* and *OsTIR1* mRNA.

*OsAFB2* is an alternatively spliced gene with at least two corresponding mRNA forms (AK072338: *OsAFB2-1* and AK100862: *OsAFB2-2*; Table 1). Both alternatively spliced mRNA transcripts (*OsAFB2-1* and *OsAFB2-2*) were downregulated in the *OsmiR393*-overexpressing rice plants, but the *OsAFB2-1* mRNA was reduced further than the *OsAFB2-2* mRNA (Figure 4). In addition, based on data analysis of the rice chip-expression database (RiceXPro: http://ricexpro.dna.affrc.go.jp/index.html), the signal intensity of the *OsAFB2-1* mRNA was much higher than that of the *OsAFB2-2* mRNA. These data indicated that *OsAFB2-1* may be the main transcript of *OsAFB2* in rice.

Both target genes (*OsAFB2* and *OsTIR1*) of *OsmiR393* were classified as auxin receptor homologs, but their functions have not yet been experimentally demonstrated in rice. Homologous analysis showed that *OsAFB2* and *OsTIR1* had a high similarity to *Arabidopsis AFB2* (At1g12820) and *TIR1* (At3g62980) (Table 1), which had been found as auxin receptors involved in primary and lateral root growth inhibition in response to nitrates [21], [22]. Spatial-expression results showed that the two genes were differentially expressed; *OsTIR1* was only expressed in the flag leaves, but *OsAFB2* was expressed in root, stems, flag leaves, panicles and shoot sheaths at the booting stage. In addition, the two alternatively spliced mRNAs of *OsAFB2* showed different expression patterns (Figure 3). One alternatively spliced mRNA (*OsAFB2-1*, AK072338) of *OsAFB2* was expressed in the stems, flag leaves and panicles, whereas the other alternatively spliced mRNA (*OsAFB2-2*, AK100862) was expressed in all tested organs (Figure 3).

### 3. Overexpression of *OsmiR393* resulted in an increase in tillers and early flowering

**Figure 5 pone-0030039-g005:**
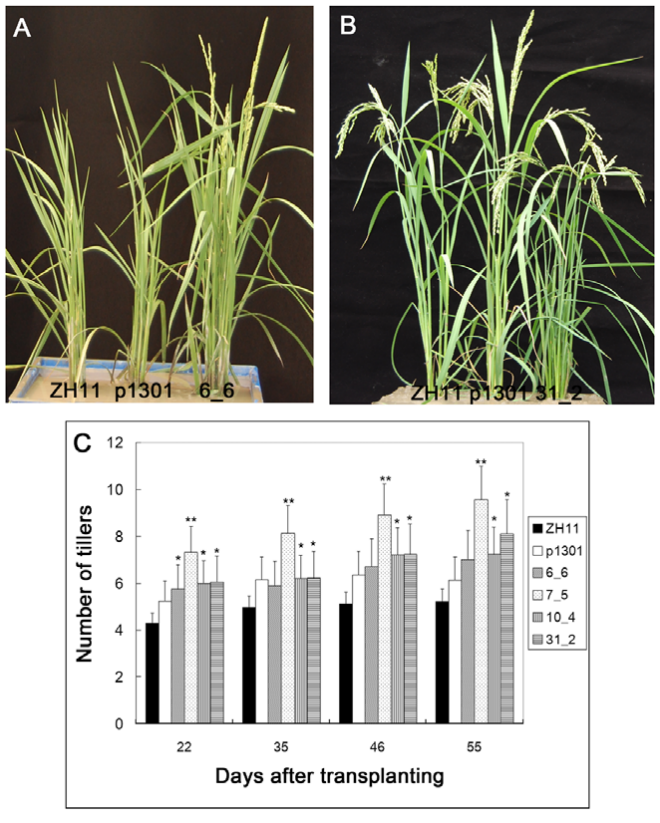
Phenotypes of the transgenic rice plants overexpressing *OsmiR393* under normal growth conditions. **A**, Early flowering (advanced one week) and **B**, increased tillers were observed in the *OsmiR393*-overexpressing rice plants. **C.** Dynamic statistics of tillers after transplanting are shown. The data were from 90 plants of three plots in the natural field; * and ** indicate significance by the Duncan's multiple range tests at the 5% or 1% level, respectively.

To investigate the newly discovered functions of *OsmiR393* in rice, we compared the *OsmiR393*-overexpressing rice plants to the wild-type rice variety Zhonghua 11 and transgenic rice plants with the empty vector pCambia1301. In the natural field, two new phenotypes (increased tillers and early flowering) in the *OsmiR393*-overexpressing rice plants were found. The flowering (heading) time of the *OsmiR393*-overexpressing rice plants was earlier by one week than the control rice plants (Figure 5A). Tillers of the *OsmiR393*-overexpressing rice plants were increased by at least 30% compared to the control plants (Figure 5B, C).

**Figure 6 pone-0030039-g006:**
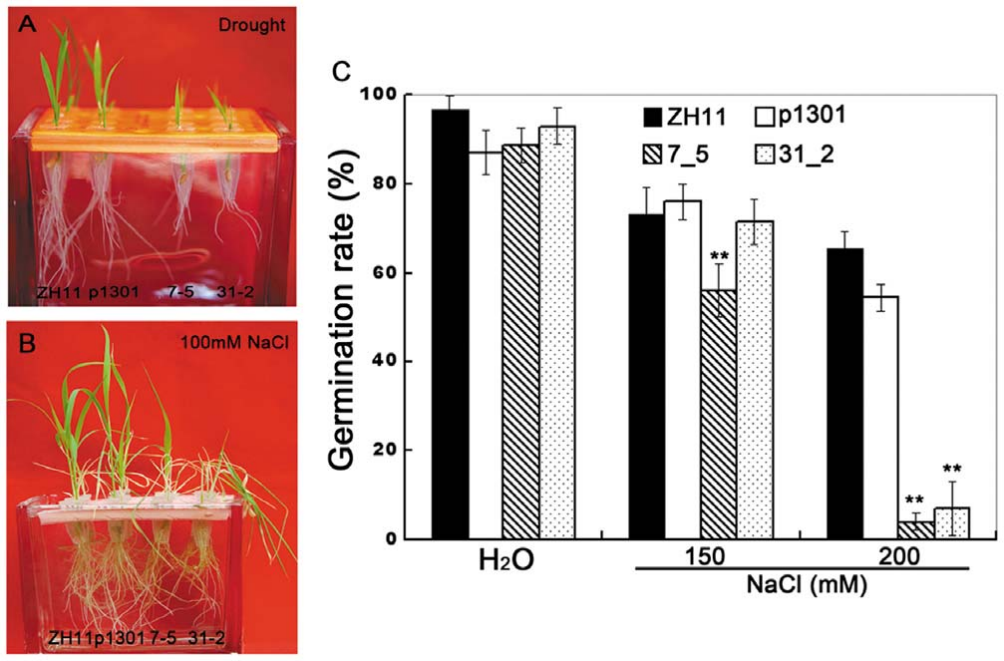
Response to drought and salt treatments. **A.** Growth was affected by drought stress. The 6-day seedlings of *OsmiR393*-overexpressing rice and controls were removed from Hoagland's Solution for one day and then re-cultured in Hoagland's Solution for 4 days. **B.** Images were taken on the fifth day after 10-day seedlings were placed in 100 mM NaCl. **C.** The germination ratio was calculated after the seeds germinated in 150 and 200 mM NaCl for 15 days; ** indicates significance by the Duncan's multiple range tests at the 1% level.

Our results also confirmed the negative role of *OsmiR393* in the tolerance of abiotic stresses, which had been found in *Arabidopsis* and rice [17], [19]. Growth of the *OsmiR393*-overexpressing seedlings was repressed by one-day drought treatments (Figure 6A). *OsmiR393*-overexpressing rice plants grew slowly compared with the control plants in nutrient solution [23] containing 100 mM NaCl (Figure 6B). Treatment with 250 mM NaCl repressed the seed germination of the *OsmiR393*-overexpressing rice compared to the controls (Figure 6C). These data indicated that the *OsmiR393* negatively regulates salt and drought tolerance.

### 4. Overexpression of *OsmiR393* repressed the auxin signaling pathway

**Figure 7 pone-0030039-g007:**
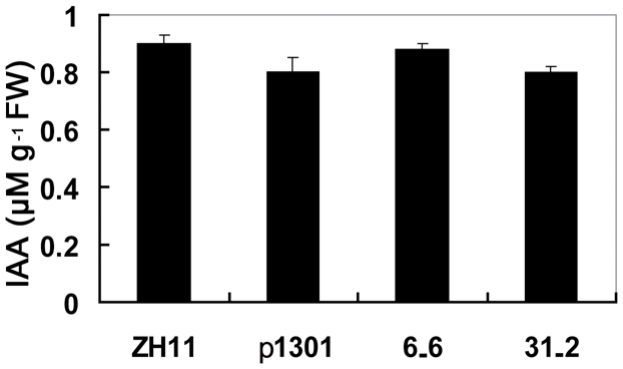
Auxin (IAA) contents in *OsmiR393*-overexpressing rice plants. The rice was grown in the field during the rice-growing season, and IAA concentrations in flag leaves were measured.

As previously documented in *Arabidopsis*, *miR393* silences its target genes *TIR1* and *AFBs*, which encode auxin receptors in *Arabidopsis* [4], [12]. To examine whether the roles of *OsmiR393* in rice are similar with *Arabidopsis* through the auxin signaling pathway, the responses of the *OsmiR393*-overexpressing rice plants to auxin were investigated. We found that overexpression of *OsmiR393* lead to a reduction in the transcript of the two auxin receptor gene homologs (Figure 4). However, the IAA content analysis in the flag leaves showed no difference between the *OsmiR393*-overexpressing rice plants and the controls (Figure 7). These data indicated that the synthesis of IAA in the *OsmiR393*-overexpressing rice plants did not change.

**Figure 8 pone-0030039-g008:**
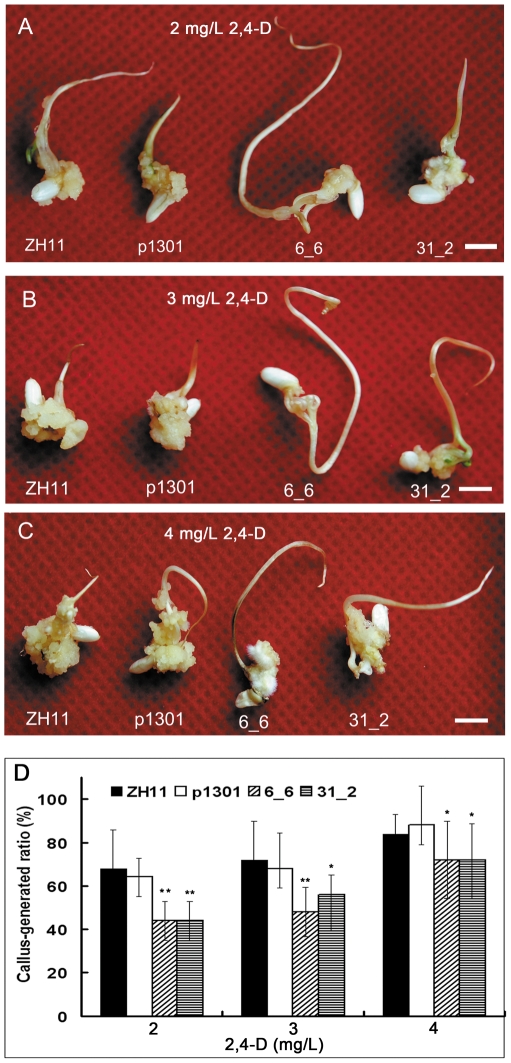
Effects of 2,4-dichlorophenoxyacetic acid (2, 4-D, a synthetic auxin analog) on callus induction. Callus induction under 2 mg/L (**A**), 3 mg/L (**B**) and 4 mg/L (**C**) of 2,4-D. Callus-inducing rates of *OsmiR393*-overexpressing rice seeds were much lower than that of controls, and they easily generated buds and roots. Bar = 0.5 cm. **D.** Statistics of callus-inducing rate after 21 days of cultivation; * and ** indicate significance by the Duncan's multiple range tests at the 5% or 1% level, respectively.

**Figure 9 pone-0030039-g009:**
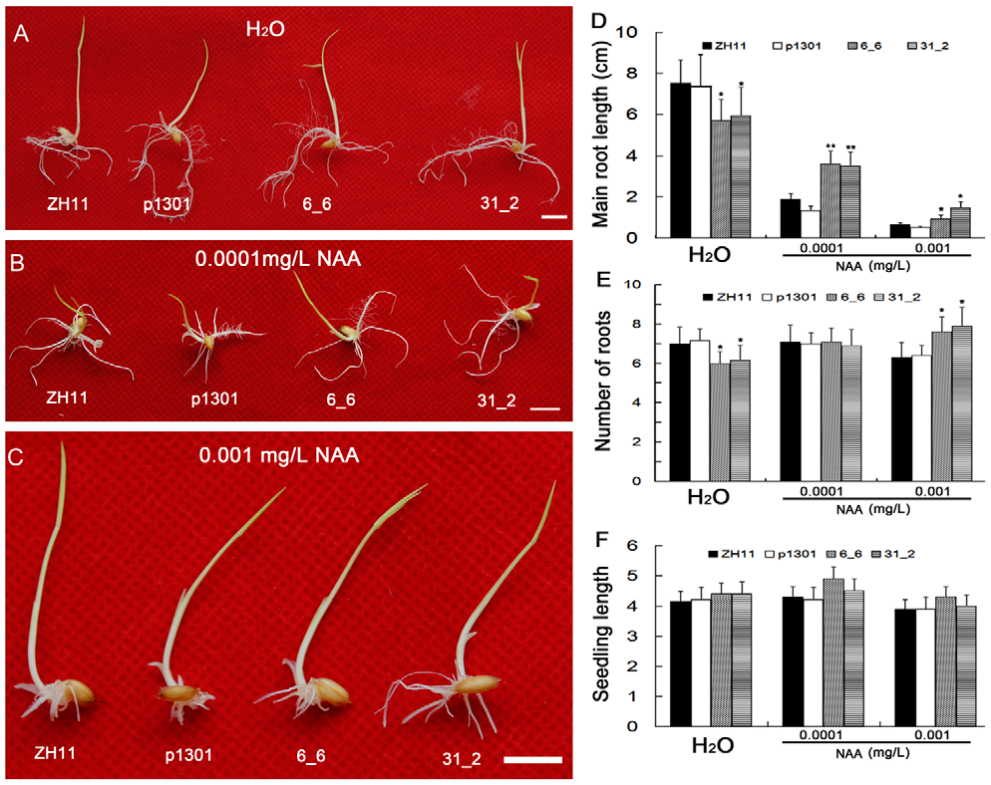
Effects of 1-naphthaleneacetic acid (NAA, a synthetic auxin analog) on root growth. Seeds were germinated in different concentrations of NAA for 10 days in the dark at 26±2°C. Bar = 1.0 cm; * and ** indicate significance by the Duncan's multiple range tests at the 5% or 1% level, respectively.

Compared to the controls, the *OsmiR393*-overexpressing rice plants exhibited auxin hyposensitivity. Mature seed callus induction of *OsmiR393*-overexpressing rice was hyposensitive to 2,4-D (Figure 8A–D). The size of the callus was smaller than the controls (Figure 8B, C) and the callus-inducing rate of *OsmiR393*-overexpressing rice seeds was lower than the controls (Figure 8D). However, no significant differences were found in the germination ratios of the *OsmiR393*-overexpressing rice and the controls under different NAA treatments (data not shown). NAA treatments exerted an influence on root growth. Under the inhibitory growth concentration of NAA, more roots (Figure 9B,C,E) and longer main roots (Figure 9B–D) of *OsmiR393*-overexpressing rice plants were observed in 0.001 and 0.0001 mg/L NAA treatments compared with controls. However, when they were germinated in water (Figure 9A), the root length and root numbers of OsmiR393-overexpressing rice plants were lower than that of the controls (Figure 9D, E). These data may indicate that the roots of the *OsmiR393*-overexpressing rice plants are less responsive to normal physiological concentrations of NAA. These responses to NAA suggested that the auxin receptors might be damaged by overexpression of *OsmiR393*.

### 5. Expression analysis of *OsAFB2* or *OsTIR1* downstream genes related to tillering and flowering time

**Figure 10:**
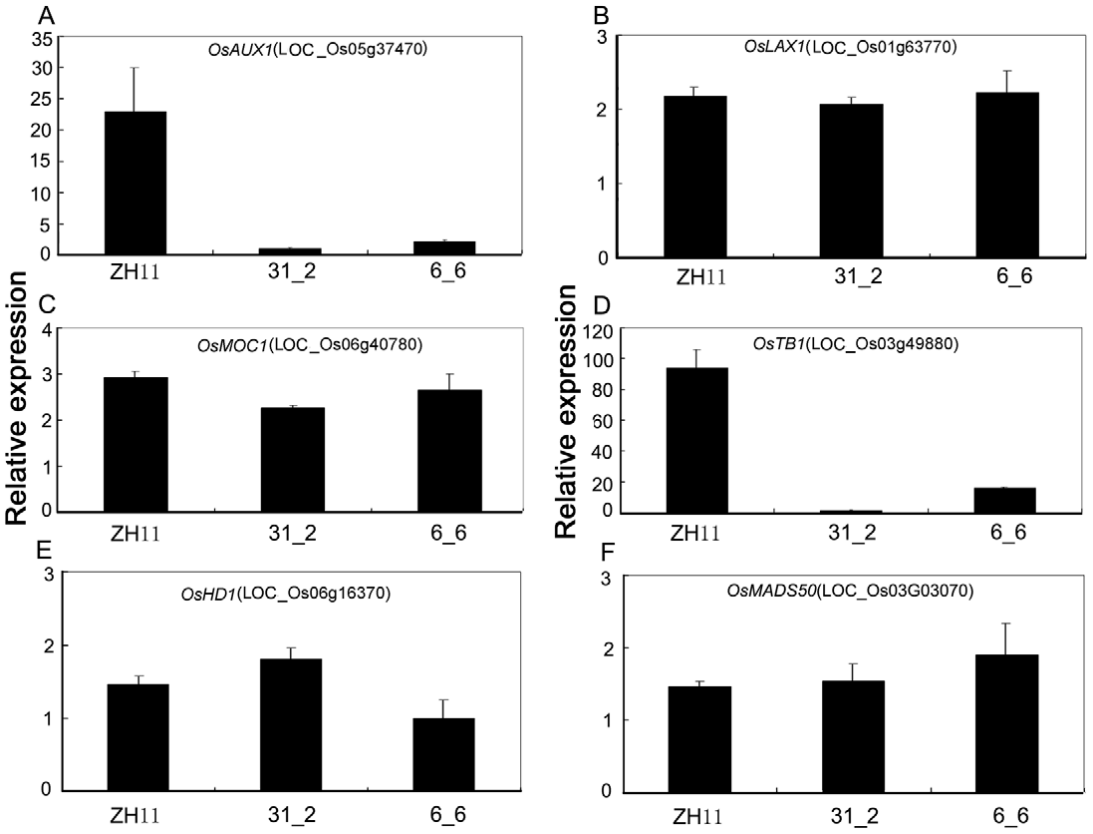
Expression comparison of genes downstream of *OsAFB2* and *OsTIR1* that control the tillers and flowering time. Total RNA was isolated from the booting rice plants grown in field. Rice *Actin1* was used as an internal control. Two auxin transporter homologs (**A, B**), an enhancing tiller gene (**C**), a tillering inhibitor (**D**) and two genes promoting flowering (**E, F**) of rice were compared.

To form a chain of *OsmiR393* on the phenotype via the auxin signaling pathway, we compared the expression of two auxin transporter genes (*OsAUX1*: LOC_Os05g37470 and *OsLAX1*: LOC_Os01g63770) [13] in *OsmiR393*-overexpressing lines and the controls using the quantitative RT-PCR. However, the expression of only one gene (*OsAUX1*) was downregulated in the *OsmiR393*-overexpressing rice (Figure 10A) compared with the controls when using a two-fold change threshold. These data indicated that *OsmiR393* could regulate the auxin transport via *OsAUX1*. To determine the mechanism by which *OsmiR393* controls tillering and flowering, the expression levels of genes related with tillering (Figure 10C, D) and flowering time (Figure 10E, F) of rice were compared among the *OsmiR393*-overexpressing lines and the controls. The data showed that overexpression of *OsmiR393* could downregulate expression levels of *OsTB1* (LOC_Os03g49880; Figure 10D), which is a negative regulator for lateral branching and inhibits tillering in rice [24]. However, *OsmiR393* did not regulate expression levels of *MOC1*, which enhances rice tillering [25]. The expression of two genes (*OsHd1* and *OsMADS50*) [26] related to flowering time was not affected by *OsmiR393* (Figure 10E, F).

## Discussion

### 1. Prediction of target genes of *OsmiR393* requires experimental confirmation

Many computational predictions of miRNA target genes have been conducted [2], [27], [28]. The microRNA *miR393* has been identified in diverse species including monocotyledons and dicotyledons [12], [17]. Two mature *miR393* isoforms, *OsmiR393* and *OsmiR393b*, arising from two primary transcripts, have been identified in rice and *Arabidopsis* (http://www.mirbase.org/cgi-bin/query.pl?terms=miR393). Previous studies indicated that *miR393* targets the auxin receptors in *Arabidopsis* and rice [4], [12]. In rice, candidate *OsmiR393*-targeted genes have been predicted [17], but they have not been experimentally investigated. Although the program miRU predicted that seven genes in the rice genome are candidate target genes of *OsmiR393* (Table 1), only two genes (*OsAFB2* and *OsTIR1*) were actually targeted by *OsmiR393* (Figure 3) in the *OsmiR393*-overexpressing rice plants. These data indicated that the target genes predicted by computational programs should be experimentally validated.

The microRNA *miR393* regulates plant growth and development by regulating target genes, which encode F-box proteins such as *TIR1*, *AFB2* and *AFB3* in *Arabidopsis* [4], [12], [29]. Those F-box proteins, especially *TIR1*, act as auxin receptors and play crucial roles in controlling the expression of genes associated with the auxin signaling pathway, which is implicated in various aspects of plant growth and development [30], [31], [32]. Our results indicated that rice *OsmiR393* could also target the mRNAs of two auxin receptor gene homologs in the overexpressing-*OsmiR393* transgenic rice plants (Figure 3), suggesting that the regulation pathway of *miR393* might be conserved among the monocot and dicot species through regulation of the transcript abundance of the auxin receptor genes. It has been previously reported that mature *miR393* sequences are a canonical microRNA conserved among different plant species such as *Arabidopsis*, rice, maize, poplar, *Medicago* and *Brassica napus* [9], [17], [33], [34], [35]. The conservation in *miR393* itself and the recognition sites of its target genes suggest that *miR393* functions in a similar way among different plants, including rice.

### 2. Functions of *OsmiR393* and its target genes

In high-yielding semidwarf rice, early flowering and high tillering capacity are considered beneficial for the grain yield of rice [1]. Several gene mutations showing a relationship to the regulation of tillering have been found in rice. Mutations in some genes decrease rice tillers including *moc1* [25] and *htd-1* [36]. Some mutations increase tillers including *Htd1* [37] and *D10* [38]. Flowering time is controlled by many quantitative trait loci in rice [39]. *Hd1* [40] and *Ghd7* [41] repress floral transition strongly under long-day (LD) conditions, but *Ehd1* promotes floral transition [42]. We found that overexpression of *OsmiR393* in rice caused significant changes in two yield-associated traits, increased tillers and early flowering (Figure 5).

*OsmiR393* overexpression reduced the mRNA abundance of *OsTIR1* and *OsAFB2* (Figure 3) but did not change the content of IAA (Figure 7). The *OsmiR393*-overexpressing rice also decreased sensitivity to two synthetic auxin analogs (Figure 8, 9). Auxin acts through the TIR1 auxin receptor protein [43]. Upon auxin binding, TIR1 recruits specific transcriptional repressors (the Aux/IAA repressors) for ubiquitination by the SCF complex [43]. This process leads to the degradation of the Aux/IAAs repressors by the proteasome. The degradation of the repressors leads in turn to the potentiation of auxin response factors and AFB-mediated transcription of specific genes in response to auxins [44]. Our results indicated that *OsmiR393* regulated the abundance of *OsTIR1* and *OsAFB2* mRNA (Figure 3), which are involved in this auxin pathway.

The two new phenotypes (increased tillers and early flowering) of *OsmiR393*-overexpressing rice plants might arise from the hyposensitivity to auxin. Auxin induces shoot apical dominance; the axillary buds are inhibited by auxin [45]. However, the *OsmiR393*-overexpressing rice plant showed more tillers (Figure 5B) indicating that the inhibitory effect is removed, and the growth of lateral buds is enhanced at a high concentration of auxin. Auxin might also play a minor role in the initiation of flowering (Figure 5A).

### 3. A gene chain model from *OsmiR393* to rice tillering via auxin signaling

**Figure 11:**
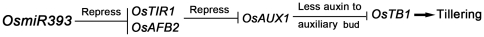
A gene chain model from *OsmiR393* to tillering via auxin signaling. Upregulation of *OsmiR393* represses expression of two auxin receptors (*OsAFB2* and *OsTIR1*). Downregulation of *OsAFB2* and *OsTIR1* then leads to reduced expression of an auxin transporter (*OsAUX1*). Low levels of OsAUX1 results in a decrease of auxin transported to the auxiliary buds. The low auxin in auxiliary buds reduces expression of a tillering inhibitor (*OsTB1*). Finally, the downregulation of *OsTB1* promotes the tillering of rice.

Similar to *Arabidopsis*, the posttranscriptional repression of *OsTIR1*/*OsAFB* genes by *OsmiR393* alters rice auxin responses (Figure 4). Here, we found that an auxin transporter gene (*OsAUX1*) and a rice tiller inhibitor gene (*OsTB1*) were repressed by the overexpression of *OsmiR393* (Figure 10). Therefore, we propose a pathway for the controlling of rice tillering by *OsmiR393* (Figure 11). *OsmiR393* represses the expression of *OsTIR1* and *OsAFB*, which further represses the expression of *OsAUX1* (Figure 10A). Therefore, less auxin is transported to the auxiliary buds resulting in low-levels of auxin in the axillary buds. The low levels of auxin in the axillary buds induce downregulation of *OsTB1* (a tillering inhibitor) [24] (Figure 10D). The downregulation of *OsTB1* promotes axillary bud development and finally results in an increase in tillers in the *OsmiR393*-overexressing rice plants (Figure 5).

### 4. Implications for rice breeding

Tillering ability and flowering time control are often associated with yield-related traits of rice; therefore, both traits are very important agronomic traits for the genetic improvement of rice. *OsmiR393* overexpression could improve the tillering and early flowering of rice (Figure 5), which are positive goals for molecular breeding of rice. However, a previous study [17] and this study also found that *OsmiR393* was negatively involved in the salt and drought stress responses. The *OsmiR393*-overexpressing rice plants were less tolerant to NaCl or drought treatments (Figure 6). These may cause a dilemma regarding the use of *OsmiR393* in rice molecular breeding, which could increase tillers and early flowering but could also impair the stress tolerance.

## Materials and Methods

### 1. Vector construction

Rice mature *OsmiR393* sequences (accession: MIMAT0000957) were downloaded from miRBase (http://www.mirbase.org). A vector with artificial microRNAs (amiRNAs) *OsmiR393* was constructed following the procedures from the website (http://wmd3.weigelworld.org/downloads/modify_pNW55.pdf) and the descriptions by Warthmann [46]. Briefly, the three resultant fragments (amplification from pNW55 with the primers designed by the website for the rice miR393 sequence) were gel purified (TransGen Biotech, Lot#E30719) and fused by a PCR reaction with the two flanking primers G-4368 (with the restriction site *Bgl*II) and G-4369 (with the restriction site *Afl*II) using 1 µl of the mixture of the three previous PCR products as a template. The fusion product of 554 bp was cloned into pGEM-T Easy Vector (Promega, USA). After verification by sequencing, the constructs were digested with *Bgl* II and *Afl* II and transferred to the binary vector pCambia1301 (pCambia, Australia) by replacing the *gus* site. In pCambia1301, the expression of the transgene is driven by the 35S CaMV promoter. The *Ami393* plant expression vector and the control vector pCambia1301 were transformed into *Agrobacterium tumefaciens* strain *EHA*105.

### 2. Rice transformation and trait records

The rice variety Zhonghua11 (*japonica*) was transformed by *Agrobacterium*-mediated transformation [20] and selected with *hygromycin*. All regenerated T_0_ transgenic plants were genotyped using the primer O27F (within the CaMV 35S promoter) and G4369 (Table S1). Plants were grown in the South China Botanical Garden fields under normal growth seasons. The number of total tillers was determined after transplanting on the indicated days, and the flowering time was compared with the controls.

### 3. Target gene analysis of *OsmiR393* with bioinformatics

The prediction of target genes was performed by the web-based prediction program miRU (Plant microRNA Potential Target Finder, http://bioinfo3.noble.org/miRNA/miRU.htm). The TIGR Rice Genome mRNA database and putative targets with an expectation score of ≤3 for each of 20 nucleotides were selected; other options were set to default. The gene expression was also analyzed in the chip-expression database (RiceXPro: http://ricexpro.dna.affrc.go.jp/index.html).

### 4. RT-PCR/qRT-PCR

#### 4.1. Small RNA extraction and reverse transcription

Small RNA was extracted from rice using an RNAiso kit for small RNA (Takara, Cat# D340A) and digested with DNase I (Takara, Code; D2215) according to the product manuals. Reverse transcription was performed with a cDNA Synthesis Kit (Promega, Cat# M1701) in combination with stem-loop RT-PCR technique [47]. Briefly, the reaction was performed with 1 µg of total RNA, 2 µL 0.05 µM RT primer mix (0.05 µM *OsmiR393* RT primer and U6 RT primer each) in a volume of 12.5 µl. The reaction was incubated at 65°C for 5 min and then in ice-water for at least 2 min to ensure the formation of stem-loops and combination with miRNA from the miRNA-Primer Mix. Then, 4 µl of the 5× M-MMLV Reverse Transcriptase buffer, 1 µl M-MMLV, 2 µl 10 mM each dNTPs, 0.5 µl RNase inhibitor (0.25 U/µl) were added to the 12.5 µL miRNA-Primer Mix for a total reaction volume of 20 µl. The reaction was incubated at 25°C for 25 min, 42°C for 30 min and 70°C for 10 min to inactivate the reverse transcriptase. To reduce the high abundance effect on PCR amplification, the reverse transcription mixture was diluted 20 times with water.

#### 4.2. RNA extraction for total RNA and reverse transcription

Total RNA was extracted from rice with Trizol (Invitrogen). The total RNA reverse transcription reaction was performed with a two-step RT-PCR kit (Promega, Cat.# M 1701) on 2 µg of total RNA according to the product manual.

#### 4.3. Expression detection by real-time quantitative PCR

Gene expression was analyzed by quantitative real time RT-PCR and semi-quantitative RT-PCR using the primers listed in Table S1. PCR amplification was performed in the presence of the double-stranded DNA-specific dye SYBR Green (Takara) and monitored in real time with the 7500 RT-qPCR system (Applied Biosystems). Rice *Actin1* (LOC_Os03g50885) served as the standard for normalizing the expression of predicted target genes in semi-quantitative RT-PCR. snRNA *U6* [48] served as a reference gene for the detection of miRNAs in quantitative real time RT-PCR.

PCR amplification reactions were performed in a volume of 20 µl containing 10 µl of TaqMan Master Mix SYBR *Premix* Ex Taq (Takara), 1 µl of 20 times diluted cDNA and 0.8 µl each of forward and reverse primer (10 µM each). Cycling conditions were as follows: 95°C for 30 sec, followed by 40 cycles of 95°C for 10 sec and 61°C for 20 sec. PCR reactions were performed on the 7500 RT-qPCR system (Applied Biosystems). Raw Cq values were calculated using the SDS software v.2.0.1 using automatic baseline settings and a threshold of 0.2.

### 5. Extraction, purification and determination of indole-3-acetic acid (IAA)

The analysis of indole-3-acetic acid (IAA) was performed according to Kuraishi et al. [49] and Ağar et al. [50]. The frozen sample (2 g) was powdered in liquid nitrogen. The isolation process for IAA followed a previously published protocol [49], [50]. The hormone extracts were injected into WATERS2695 High Performance Liquid Chromatography (HPLC, American) to measure IAA. A standard IAA sample was obtained from Sigma-Aldrich (St. Louis, MO).

### 6. Effect of 2,4-dichlorophenoxyacetic acid (2, 4-D) on callus induction

Sterilized seeds of rice were inoculated on MS medium containing 2, 3 and 4 mg/L of 2,4-D [51]. The cultures were incubated at 26±2°C in the dark. After 21 days of inoculation, callus generation rates induced from the different mature seeds were calculated and images were acquired.

### 7. Response of seeds and seedlings to auxin treatments

The seeds were soaked in 0, 0.001 and 0.0001 mg/L NAA overnight. Subsequently, the soaked seeds were transferred to a petri dish with a filter paper soaked with different concentration of NAA with high humidity and then incubated in the dark at 26±2°C. After 10 days of germination and growth, root numbers, root length and seedling length were measured and images were acquired.

### 8. Salt and drought treatments

To examine the effects of salt stress on germination, the rice seeds were germinated in NaCl solution for 15 days. The seedlings (3 mm in length) were calculated as the germinated seeds. For drought treatment, the 6-day seedlings were removed from the Hoagland's Solution [23] for 1 day and then re-cultivated in Hoagland's Solution for 4 days.

### 9. Statistical analysis

All the data were analyzed from at least 30 plants for each trait with SAS software. Statistical analysis was performed with Duncan's multiple range tests.

## Supporting Information

Figure S1 **Monitor cleavage of** ***OsAFB2*** **and** ***OsTIR1*** **mRNA by 5′-RACE.** Total RNAs were extracted from the wild type rice ZH11 and *OsmiR393* overexpressing line. **A.** The target site schematic diagram of *OsAFB2* and *OsTIR1* mRNAs by *OsmiR393* and the expected sizes of 5′-RACE with their gene specific primers. **B.** Image of 5′-RACE.
(TIF)

Table S1 **Primer sequences used in this study.**
(DOC)
