# Supplementary figures and images for: OsTIR1 and OsAFB2 Downregulation via OsmiR393 Overexpression Leads to More Tillers, Early Flowering and Less Tolerance to Salt and Drought in Rice
Source: PLoS One. 2012 Jan 10;7(1):e30039. doi: 10.1371/journal.pone.0030039 (PMC3254625; doi:10.1371/journal.pone.0030039)

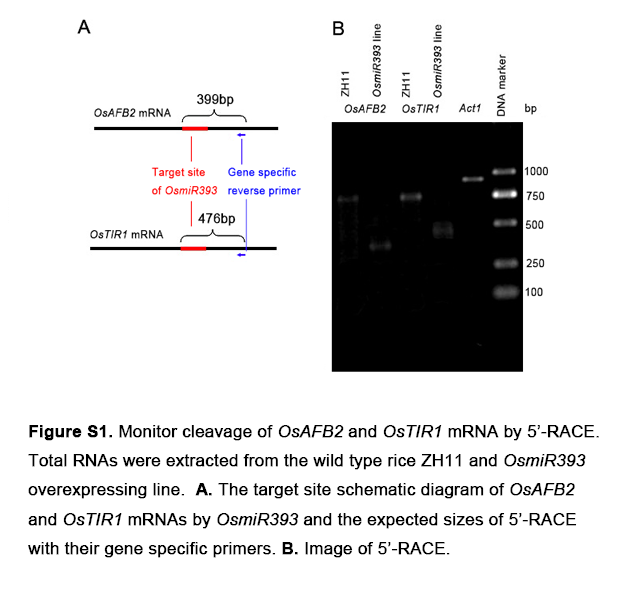

Supplement: Figure S1 — Monitor cleavage of OsAFB2 and OsTIR1 mRNA by 5′-RACE. Total RNAs were extracted from the wild type rice ZH11 and OsmiR393 overexpressing line. A. The target site schematic diagram of OsAFB2 and OsTIR1 mRNAs by OsmiR393 and the expected sizes of 5′-RACE with their gene specific primers. B. Image of 5′-RACE. (TIF) [file pone.0030039.s001.tif]
