# Supplementary material for: OsTIR1 and OsAFB2 Downregulation via OsmiR393 Overexpression Leads to More Tillers, Early Flowering and Less Tolerance to Salt and Drought in Rice
Source: PLoS One. 2012 Jan 10;7(1):e30039. doi: 10.1371/journal.pone.0030039 (PMC3254625; doi:10.1371/journal.pone.0030039)
Supplement: Table S1 — Primer sequences used in this study. (DOC) [file pone.0030039.s002.doc]

**Supplementary Table 1** Primer sequences used in this study

| **Name** | **Sequence (5' - 3')** | **Note** |
| --- | --- | --- |
| G-4368 | GAAGATCTCTGCAAGGCGATTAAGTTGGGTAAC | Insert restriction site *Bg*l II |
| G-4369 | CCCCTTAAGGCGGATAACAATTTCACACAGGAAACAG | Insert restriction site *Afl*II |
| 393 I miR-s | agTCCAAAGGGATCGCATTGATCCcaggagattcagtttga | *OsmiR393* replace miR528 in pNW55 vector |
| 393 II miR-a | tgGGATCAATGCGATCCCTTTGGActgctgctgctacagcc | *OsmiR393* replace miR528 in pNW55 vector |
| 393 III miR*s | ctGGATCTATGGGATCCCTTTGGAttcctgctgctaggctg | Reverse complementary *OsmiR393* replace miR528 in pNW55 vector |
| 393 IV miR*a | aaTCCAAAGGGATCCCATAGATCCagagaggcaaaagtgaa | Reverse complementary *OsmiR393* replace miR528 in pNW55 vector |
| O27 F | ATCCTTCGCAAGACCCTTCCTC | Location in 35s promoter |
| OsAFB2-1 q-F | GAGCGGGATGGTAGCAATGAAATG | q-PCR for mRNA (AK072338) of LOC_Os04g32460 |
| OsAFB2-1 q-R | CCGAGATAAGGGAGGCACACCAAc |
| OsAFB2-2 q-F | AAATGAGCGGGATGGTAGCAATGAAATG | q-PCR for mRNA (AK100862) of LOC_Os04g32460 |
| OsAFB2-2 q-R | TAATGTATTGAAAGGGGTTACCTTATGG |
| 36080.1 q-F | TCAAGGAGGACCAGCAGTTCACCA | q-PCR for mRNA of LOC_Os03g36080 |
| 36080.1 q-R | GAGGACACCCGTGCTGACCAAAGT |
| 41010.1 q-F | GCGGAGGTGGAGGCGGGAAGG | q-PCR for mRNA of LOC_Os05g41010 |
| 41010.1 q-R | CGCGAGGGAGACGAGGTGGTTG |
| OsMGT6 q-F | TATGGTATGCCCGGTGGAAGAAGT | q-PCR for mRNA of LOC_Os10g39790 |
| OsMGT6 q-R | ATACTGCTGCCTCAGGATGATTGG |
| OsGRF1 q-F | TGCTTCCCCTACCACCGTCACCAC | q-PCR for mRNA of LOC_Os03g52320 |
| OsGRF1 q-R | GCGCACTCTTCCACCTTCCCATTG |
| 58734.1 q-F | TTGCTTCCGCCTCCTCATCTTCCT | q-PCR for mRNA of LOC_Os04g58734 |
| 58734.1 q-R | GCCGTGCGAACCATATTCCTTTGA |
| OsTR1 q-F | TCCAGGTGCTCCGCCTCGTCTCCT | q-PCR for mRNA of LOC_Os05g05800 |
| OsTR1 q-R | CCGGGAAGAGGCTGAGCCAATGAA |
| 393-RT | GTCGTATCCAGTGCAGGGTCCGAGGTATTCGCACTGGATACGACGATCA | For reverse transcript of *OsmiR393* |
| U6-RT | ATTTGGACCATTTCTCGATTTGT | For reverse transcript of snRNA *U6* [48] |
| 5800.1 -RT-F | AGCTTCAAGCGCATGGTCGT | For semi-quantitative RT-PCR of LOC_Os05g05800 |
| 5800.1-RT-R | GTGCCTCATTTGTCATCCGTCT |
| 32460.1-RT-F | TCAGGATGAAGCGGATGGTG | For semi-quantitative RT-PCR of LOC_Os04g32460 |
| 32460.1-RT-R | CTTCCCTGGCTCAAGAATACAAA |
| 393-q-F | GACTGTCCAAAGGGATCGCATT | For q-PCR of *OsmiR393* |
| 393-q-R | GTGCAGGGTCCGAGGTATTC |
| Actin1 q-F | CGGTGTCATGGTCGGAAT | For q-PCR of *Actin1* (LOC_Os03g50885) |
| Actin1 q-R | GCTCGTTGTAGAAGGTGT |
| U6 q-F | CGATAAAATTGGAACGATACAGA | For q-PCR of snRNA *U6* [48] |
| U6 q-R | ATTTGGACCATTTCTCGATTTGT |
| MOC1-q-F | GGGGGTGTGCTTGTTCTTGTTG | q-PCR for MOC1（Os06t0610300-01） |
| MOC1-q-R | GTTTGGACGGCTGCTCTGTGTTC |
| OsTB1-q-F | GGGAGGCCAGCGAGCAGCAGTAGC | q-PCR for OsTB1 (LOC_Os03g49880) |
| OsTB1-q-R | AGGGGAAGGAAGGGGAGGGAGGTG |
| OsAUX1-q-F | TTCCTCGCCATCATCTTCCCCTT | q-PCR for mRNA of LOC_Os05g37470 |
| OsAUX1-q-R | TTCACCACGAACATCCCGCTC |
| OsLAX1-q-F | TACTTCGTGTGGGAGAAGGTGAT | q-PCR for mRNA of LOC_Os01g63770 |
| OsLAX1-q-R | TACGGTAGGTGAGGATGTGGG |
| OsMADS50-q-F | TGGACCGCCTACCTCATCAGCAT | q-PCR for mRNA of LOC_Os03g03070 |
| OsMADS50-q-R | TCACCGACCCGAACAGCAAAAAC |
| Hd1-q -F | TCAGCAACAGCATATCTTTCTCATCA | q-PCR for mRNA of LOC_Os06g16370 |
| Hd1-q-R | TCTGGAATTTGGCATATCTATCACC |
